# Engineered AAV2.7m8 Serotype Shows Significantly Higher Transduction Efficiency of ARPE-19 and HEK293 Cell Lines Compared to AAV5, AAV8 and AAV9 Serotypes

**DOI:** 10.3390/pharmaceutics16010138

**Published:** 2024-01-19

**Authors:** Dzerassa Gurtsieva, Ekaterina Minskaia, Sofia Zhuravleva, Elena Subcheva, Elena Sakhibgaraeva, Andrew Brovin, Artem Tumaev, Alexander Karabelsky

**Affiliations:** Department of Gene Therapy, Sirius University of Science and Technology, Olympic Avenue, 1, 354340 Sochi, Russia; gurtsieva.dt@talantiuspeh.ru (D.G.); zhuravlyova.sv@talantiuspeh.ru (S.Z.); subcheva.en@talantiuspeh.ru (E.S.); sahibgaraeva.el@talantiuspeh.ru (E.S.); brovin.an@talantiuspeh.ru (A.B.); tumaev.av@talantiuspeh.ru (A.T.); karabelskiy.av@talantiuspeh.ru (A.K.)

**Keywords:** IRD, retinopathy, gene therapy, AAV serotypes, AAV tropism, retina, AAV2.7m8, RDH12 deficiency, ARPE-19

## Abstract

The level of transduction efficiency of the target retinal cells affects the choice of AAV serotype and the outcome of gene replacement therapy for inherited retinal diseases. This study focused on the tropism and transduction efficiency of AAV2.7m8-, AAV5-, AAV8-, and AAV9-GFP in ARPE-19 and HEK293 cells. Fluorescence intensity was assessed bi-hourly by means of IncuCyte S3 live imaging microscopy. Within 12 h, AAV2.7m8 demonstrated the highest transduction efficiency at four viral concentrations of 1-, 3-, 6-, and 8 × 10^4^ VG/cell in a dose-dependent manner, followed by AAV5 in ARPE-19 and AAV9 in HEK293 cells. The transduction efficiency of AAV2.7m8 at a dose of 6 × 10^4^ VG/cell was 21, 202, and 323 times higher in ARPE-19 cells and 324, 100, and 52 times higher in HEK293 cells compared to AAV5, AAV8, and AAV9, respectively. This trend remained for 4 days at all viral concentrations, as additionally shown by flow cytometry. At a dose of 6 × 10^4^ VG/cell, AAV2.7m8 (97% GFP-positive cells, GFP +) was nearly two and 10 times as efficient as AAV5 (52% GFP+) and AAV9 or AAV8 (both 9%), respectively, in ARPE-19 cells. In HEK293 cells, 95% of AAV2.7m8-, 26% of AAV9-, 17% of AAV8-, and 12% of AAV5-transduced cells were GFP-positive.

## 1. Introduction

Inherited retinal diseases (IRDs) are a group of rare, congenital, phenotypically and genotypically heterogeneous ocular diseases characterized by severe visual impairment and blindness acquired from early to middle age. The various IRDs are caused by mutations in genes encoding the visual phototransduction cascade enzymes in the photoreceptor and neuronal cells of the retina [1,2]. The basic mechanism of pathogenesis involves the gradual die-off of photoreceptor and/or neuronal cells in the retina, leading to photophobia, loss of visual acuity, night blindness, loss of color vision, loss of central vision or tunnel vision, and eventually blindness. IRDs are the leading cause of blindness in children and working-age adults (1 in 2000 people, or more than 2 million people worldwide) [3]. Up to 836 genes listed on RetNet have been associated with IRDs. More than 20 phenotypes have been described so far and include macular, cone, cone-rod, rod-column, and chorioretinal dystrophies, cone and rod dysfunction syndromes, Leber congenital amaurosis, and Leber neuropathies [4]. Unfortunately, there is no treatment that can halt the rapid decline in vision or regenerate lost retinal cells.

Gene replacement therapy is a promising approach when applied to the loss-of-function genotypes in autosomal recessive and autosomal dominant haploinsufficiency-induced IRDs. The correct therapeutic window is critical because viable retinal cells are required for this approach [5]. Adeno-associated virus (AAV) 2-based Luxturna (voretigene neparvovec) received FDA (2017) and EMA (2018) approval as the first ocular gene therapy for the treatment of RPE65-associated retinal dystrophy. Gene therapy approaches targeting other types of IRDs, especially RDH12 (retinol dehydrogenase 12)-associated retinal diseases, for which there is no available treatment, are being developed and already demonstrating promising results [6,7]. RDH12 is one of the twenty NADPH-dependent dehydrogenase reductases present in retinal cells. Loss-of-function mutations in the *RHD12* gene lead to insufficient or a lack of RDH12 expression, leading to the accumulation of toxic intermediates of the visual cycle and lipid peroxidation in retinal cells, which, in turn, results in retinal cell apoptosis and disease progression [8]. Various approaches for the treatment of different types of Leber congenital amaurosis (LCA) and other IRDs include subretinal transplantation of stem and photoreceptor progenitor cells for the treatment of age-related macular degeneration (AMD) [9], the combination of AAVs with light-sensitive molecules for the transmission of stimulation and nerve impulse in residual retinal cells [10], viral vector-based CRISPR/Cas9 genome editing for GUCY2D autosomal dominant cone-rod dystrophy (CORD6) and the LCA type 1 gene [11], RNA editing for IRDs caused by point mutations in large genes that are not suitable for AAV-mediated gene replacement [12], and gene replacement therapy that delivers a functional copy of the missing/dysfunctional gene into retinal cells.

AAV-based gene replacement therapy, the most promising approach for the treatment of IRDs so far, demonstrates obvious advantages due to the relative simplicity and established technologies of AAV virus production, serotype-specific targeted delivery to organs and tissues, the possibility of reducing the immune response by carrying out subretinal and intravitreal injections instead of intravenous ones, and stable transgene expression in retinal cells. Intravitreal and subretinal (the second-most used route of administration) injections require the lowest average dose (8.4 × 10^10^ and 4.1 × 10^11^ viral genomes (VG)/eye, respectively). Improved vector design, the use of either suspension cells or stable cell lines as an alternative to transient transfection, and improved virus purification methods allow for the administration of higher viral doses required for systemic administration with fewer unwanted anti-capsid immune responses [13].

The optimization of AAV capsid proteins and their tropism directly affects transduction efficiency and specificity to a particular cell type [14]. The determination of the level of transduction efficiency of human retinal cells is the key to selecting the most efficient AAV serotype for gene replacement therapy for IRDs. The AAV2 serotype was successfully used for the treatment of LCA 2 (Luxturna); AAV8 and AAV5 demonstrated high tropism to retinal cells; synthetic AAV2.7m8 efficiently transduced a variety of cell types when injected subretinally [15] and also demonstrated high transduction efficiency of human iPSCs and iPSC-derived RPE cells [16]; while the self-complementary scAAV1 and scAAV2 were highly efficient in the ARPE19 and 661W cell lines [17].

This study focused on the comparison of transduction efficiency of the three natural AAV serotypes AAV5, AAV8, and AAV9 and the synthetic serotype AAV2.7m8, expressing GFP in two models: HEK293 and ARPE-19, the more relevant retinal pigment epithelium cell line, which will be used for in vitro potency tests of the newly developed gene replacement therapies for the treatment of IRDs, including RDH12-associated retinopathy. The AAV was used at four concentrations: 1-, 3-, 6-, and 8 × 10^4^ VG/cell, and the intensity of GFP expression was assessed bi-hourly by means of IncuCyte S3 live imaging microscopy, followed by flow cytometry analysis at 4 days post-transduction.

## 2. Materials and Methods

### 2.1. Cell Lines

Suspension HEK293 cells were maintained in serum-free BalanCD HEK293 media (Irvine Scientific, Santa Ana, CA, USA). The HEK293 cell line was purchased from ECACC and ARPE-19 from IBR RAS. HEK293 cells were maintained in DMEM High Glucose (4500 mg/L), 2.5 mM L-glutamine, and 10% FBS. ARPE-19 cells were maintained in DMEM/F12 (2.5 mM L-glutamine, 1× NEAA, and 10% FBS). Both adherent cell lines were maintained in a humidified incubator at 37 °C and 5% CO_2_ and grown to 80% confluence.

### 2.2. Production of Recombinant AAV2.7m8-GFP, AAV5-GFP, AAV8-GFP, and AAV9-GFP Viruses

Plasmids pAAV-RC2/9, pAAV-RC2/8, 7M8 (all from AddGene, Watertown, MA, USA), pAAV-RC2/5, pHelper, and pAAV-GFP (all from Cell Biolabs Inc., San Diego, CA, USA) were used to produce recombinant AAV of the four serotypes. pAAV-Rep/Cap plasmids contained the same Rep but different Cap sequences encoding AAV2.7m8 (p7M8), AAV5 (pAAV-RC2/5), AAV8 (pAAV-RC2/8), and AAV9 (pAAV-RC2/9) capsids for the packaging of different recombinant AAV serotypes. The plasmids were checked by means of restriction analysis and sequencing.

Suspension HEK293 cells were seeded at 5 × 10^5^ cells/mL in 150 mL of serum-free BalanCD HEK293 media (Irvine Scientific, Santa Ana, CA, USA). Twenty-four hours later, the cells were transfected with three plasmid vectors: a transfer plasmid carrying green fluorescent protein (pAAV-GFP), a plasmid encoding AAV capsid proteins (pRep/Cap), and a helper plasmid (pHelper) encoding proteins essential to viral genome replication (plasmid molar ratio of 2:2:5, respectively, 1.5 μg DNA per 1 × 10^6^ viable cells), with transfection reagent polyethylenimine (PEI) at a DNA:PEI ratio of 1:5. One day after transfection, BalanCD HEK293 Feed was added to a final concentration of 5%, and incubation continued for 5 days on an INFORS HT Multitron orbital shaker-incubator (INFORS, Bottmingen, Switzerland) at +37 °C, 5% CO_2_, 80% humidity, 100 rpm. Tween-20 was added to the cell suspension to a final concentration of 0.05%, and incubation continued for 1 h, after which benzonase (Dia-M, Moscow, Russia) and MgCl_2_ were added at 30 units/mL and 1 mM, respectively, and incubation continued for an extra hour. The lysate was centrifuged at 3000 g for 10 min and filtered after the addition of diatomaceous earth (at 1 g/100 mL of supernatant) through a disposable Stericup vacuum filtration system (Merck Millipore, Darmstadt, Germany) with a 0.22 μm membrane. The filtrate was then concentrated using a Vivaflow 200 HY ultrafiltration system (Sartorius, Göttingen, Germany) to a volume of 50 mL.

For affinity chromatography, POROS ™ CaptureSelect ™ AAVX affinity resin (Thermo Fisher Scientific, Waltham, MA, USA) was used for AAV5, AAV8, and AAV9, and AVB Sepharose High Performance (Cytiva, Marlborough, MA, USA) was used for AAV2.7m8. An equilibration buffer (50 mM Tris-HCl, 0.05% Tween 20, 0.015 M NaCl, pH 8.0 for serotypes AAV5, AAV8, and AAV9; 20 mM Tris-HCl, 0.2 M NaCl, pH 7.8 for AAV2.7m8) was applied prior to the sample loading, followed by the wash step with wash buffer (20 mM Tris-HCl, 0.05% Tween 20, pH 8.0). Elution of AAV5, AAV8, and AAV9 viruses was carried out with the elution buffer (0.1 M Glycine-HCl, 0.05% Tween 20, 0.5 M L-Arginine, pH 2.0 or 0.1 M Glycine-HCl, 0.5 M NaCl, 0.5 M L-Arginine, pH 3.5) for AAV2.7m8, and the pH of the eluate was adjusted to 7.0. The sorbent was regenerated with the regeneration buffer (8M urea, pH 1.5 for AAV5, AAV8, and AAV9, and 0.1 M citrate, pH 2.1 for AAV2.7m8). The column was preserved with 20% ethyl alcohol.

All viral eluates obtained from chromatographic purification were concentrated and buffer exchanged using an ultrafiltration system with a nominal molecular weight cutoff of 100 kDa Vivaspin 50 (Sartorius, Göttingen, Germany). Concentration was carried out to 1–2 mL in PBS buffer (0.37 M NaCl, 0.001% Pluronic F-68). The obtained samples were sterilized using a 0.22 µm syringe filter (TPP, Schaffhausen, Switzerland), stored at +4 °C, analyzed by analytical methods, and used for transduction. The viral titer, assessed by RT-PCR, was in the range of 1 × 10^11^–1 × 10^13^ VG/L (viral genomes per liter of culture). The schematic process of AAV production and analysis is shown in Figure S1.

### 2.3. Determination of the Viral Titer by RT-qPCR

Genomic titers of rAAV serotypes were determined by quantitative PCR (qPCR) as previously described [18]. The Biomaster HS-qPCR-Hi-ROX mix (Biolabmix LLC, MHR020-2040) contained ITR sequence-specific forward (5′-GGAACCCCTAGTGATGGAGTT-3′) and reverse (5′-CGGCCTCAGTGAGCGA-3′) primers and a fluorescent probe (5′-FAM-CACTCCCTCTCTGCGCGCTCG-BHQ1-3′). The components were mixed at the following ratio: 7.5 μL of Biomaster HS-qPCR-Hi-ROX mix, 1 μL H_2_O, 50 μM each oligonucleotide, and 5 μL DNA. Prior to analysis, the samples containing rAAV were treated with DNase I (Biolabmix LLC, EM-250) according to the manufacturer’s instructions, mixing 2 μL of the sample with two units of DNase I and a one-time buffer (100 mM Tris-HCl (pH 7.0), 30 mM MgCl_2_, 30 mM CaCl_2_) in a final volume of 20 μL, followed by a 30 min incubation at +37 °C and enzyme inactivation for 15 min at +55 °C to remove non-viral DNA. Next, 20 μL of Proteinase K diluted to a concentration of 0.1 U/μL was added to the sample and incubated for 1 h at +55 °C following enzyme inactivation at +95 °C for 5 min. The resulting DNA preparation was used for qPCR analysis using a standard curve. A linearized pAAV-GFP plasmid was used to construct the standard curve. A series of serial dilutions were made starting with 1.8 ng/μL (corresponding to 0.2 × 10^8^ gene copies/μL) in 10-fold increments down to 0.02 fg/μL (corresponding to 0.2 × 10^4^ gene copies/μL), the lower limit of detectable concentrations. PCR analysis was carried out using a StepOnePlus device (Thermo Scientific, Waltham, MA, USA) using the Quantification-Standard Curve application of StepOneSoftWare V2.3 software. The standard curve was considered reliable if the coefficient of determination (R2) exceeded 0.99 and the reaction efficiency was in the range of 85 to 100%. Reliable concentration measurements were made in the range from the seventh to the twenty-fifth cycle, which corresponds to intermediate and boundary points of the standard curve in the range of determined concentrations.

### 2.4. Sample Analysis Using Dynamic Light Scattering (DLS) and Size-Exclusion Chromatography (SEC) Methods

Dynamic light scattering (DLS) is a non-contact method that applies the light scattering effect and is designed to measure the size of nano- and submicron particles of a dispersed phase that have Brownian motion. The DLS method possesses an advantage over other optical methods by allowing the sample to be measured in its native form. The samples were measured at +25 °C on a Zetasizer Ultra (Malvern Panalytical Ltd., Malvern, UK) analyzer equipped with a He-Ne laser with a wavelength of 633 nm and a maximum power of 10 mW. The multi-angle light scattering method, based on the sequential capture of the analytical signal from three detection angles of scattered radiation, allowed for the estimation of the hydrodynamic diameter, the modality of particle distribution, and the fractional ratios. The small-volume quartz cuvette (Malvern Panalytical Ltd., Malvern, UK) was used for measurements. The data were processed using ZS XPLORER software v3.1.0 (Malvern Panalytical Ltd., Malvern, UK).

The relative content of AAV monomers, high molecular weight substances (HMWS), and low molecular weight substances (LMWS) were determined by means of exclusion chromatography (SEC) using a Vanquish Flex liquid chromatograph (Thermo Fisher Scientific, Waltham, MA, USA). The fractions were separated in isocratic mode using an XBridge Protein BEH SEC chromatography column, 450 Å, 2.5 μm, 4.6 mm × 300 mm (Waters, Milford, MA, USA) and mobile phase: 20 mM Na_2_HPO_4_, 150 mM KCl, pH 7.0, at a flow rate of 0.5 mL/min. The detection was performed using a fluorimetric detector with an excitation wavelength of 280 nm and an emission wavelength of 350 nm.

### 2.5. Transmission Electron Microscopy (TEM)

Transmission electron microscopy was used for the observation of rAAV morphology. A total of 10 µL of viral suspension was applied to the freshly glow-discharged copper grids (200 mesh, formvar-carbon coated (EMCN, Beijing, China) for 2 min, washed with bidistilled water, and stained with 1 droplet (10 µL) of a 1% (*w*/*v*) water solution of uranyl acetate (Polysciences Inc., Warrington, PA, USA) for 1 min. The grids were observed with the transmission electron microscope JEM2100 Plus (JEOL, Tokyo, Japan), operating at 160 kV. At least 15 grid squares were examined thoroughly, and representative micrographs were taken at the same magnifications.

### 2.6. Transduction of HEK293 and ARPE-19 Cells

Transduction with AAV2.7m8, AAV5, AAV8, and AAV9 recombinant viruses, delivering GFP reporter protein, was carried out at four concentrations: 1-, 3-, 6-, and 8 × 10^4^ VG/cell. For this purpose, 1.5 × 10^5^ HEK293 and ARPE-19 cells (per well of a 12-well plate) were transduced with rAAV-GFP viruses of different serotypes in a final volume of 1 mL per well. Both cell lines were transduced in media supplemented with 5% FBS. Twenty-four hours post-transduction, the media was changed to a new media supplemented with 2% FBS.

### 2.7. Live Imaging Microscopy IncuCyte S3

After transduction, live imaging was carried out using the IncuCyte S3 system (Sartorius) in the bright field and GFP channels (300 ms exposure time), taking images every 2 h for 4 days. The intensity of GFP expression was presented as Total Green Objects Area (µm^2^/image) (TGOA) values.

### 2.8. Flow Cytometry

Ninety-six hours post-transduction, the number of GFP-positive cells was analyzed by flow cytometry. Briefly, the cells were trypsinized, washed twice with 500 μL PBS, and resuspended in 250 μL chilled FACS buffer (1 × PBS, 2% FBS, 1 mM EDTA). The data was recorded on the CytoFLEX B2-R2-V0 flow cytometer (Indianapolis, IN, USA) using CytExpert software v1.2 gating on single FITC-positive live cells. The analysis of median fluorescent intensity (MFI) values of GFP-positive populations was carried out using FlowJo™ v10 software. 

### 2.9. Statistical Analysis

Statistical analysis of the normal distribution of samples and the confidence interval of differences for rejection of the null hypothesis was performed using GraphPad Prism 8.2.1 software (Shapiro-Wilk test, ordinary one-way ANOVA, Sidak’s multiple comparison test). Results are presented as mean ± standard deviation of 2–3 biological replicates, confidence interval: level of difference is significant (**)–*p*-val < 0.01, (***) *p*-val < 0.001, (****) *p*-val < 0.0001, not significant (ns)–*p* > 0.05.

## 3. Results

### 3.1. Production of Recombinant AAV2.7m8-GFP, AAV5-GFP, AAV8-GFP, and AAV9-GFP Viruses

The first aim of the study was to produce and purify high-quality recombinant viruses (all steps of recombinant viral production are shown in Figure S1). Briefly, six days post-transfection of suspension HEK293 cells with plasmids necessary for rAAV production, supernatants containing rAAV particles were concentrated by ultrafiltration, purified by affinity chromatography (Figure S2), and further concentrated. The obtained recombinant viruses were analyzed by means of dynamic light scattering (DLS), size-exclusion chromatography (SEC), and transmission electron microscopy (TEM) methods. The results of the dynamic light scattering analysis (Figure S3) demonstrated the presence of AAV5-GFP, AAV8-GFP, AAV9-GFP, and AAV7m8-GFP particles with hydrodynamic diameters of 29.6 nm, 28.3 nm, 28.8 nm, and 26.6 nm at a concentration of 2.5 × 10^13^, 3.2 × 10^13^, 5.0 × 10^13^, and 8.4 × 10^11^, respectively. The average volume fraction of particles was 99.99%. Sample analysis by means of size-exclusion chromatography revealed that the relative monomer percentage for AAV5-GFP, AAV8-GFP, AAV9-GFP, and AAV7m8-GFP samples was 95.9%, 99.4%, 99.2%, and 79.8%, while the aggregate fraction remained at 4.1%, 0.6%, 0.8%, and 0.9%, respectively (Figure S4). The results of transmission electron microscopy of the virus preparations are presented in Figure S5. The studied viral particles had pronounced axes of symmetry and corresponded to the shape of an icosahedron. No impurities were found in the samples. Individual agglomerates came into view.

Genomic titers of the four rAAV serotypes were in the range of 1 × 10^11^–1 × 10^13^ VG/L (viral genomes per liter of culture), as determined by RT-qPCR. Total viral particle concentrations were 1.6 × 10^12^ (AAV2.7m8-GFP), 6.6 × 10^11^ (AAV5-GFP), 4.3 × 10^12^ (AAV8-GFP), and 2.3 × 10^12^ (AAV9-GFP) VG/mL.

### 3.2. Superior Transduction Efficiency by AAV2.7m8 at 12 h Post-Transduction

**Figure 1:**
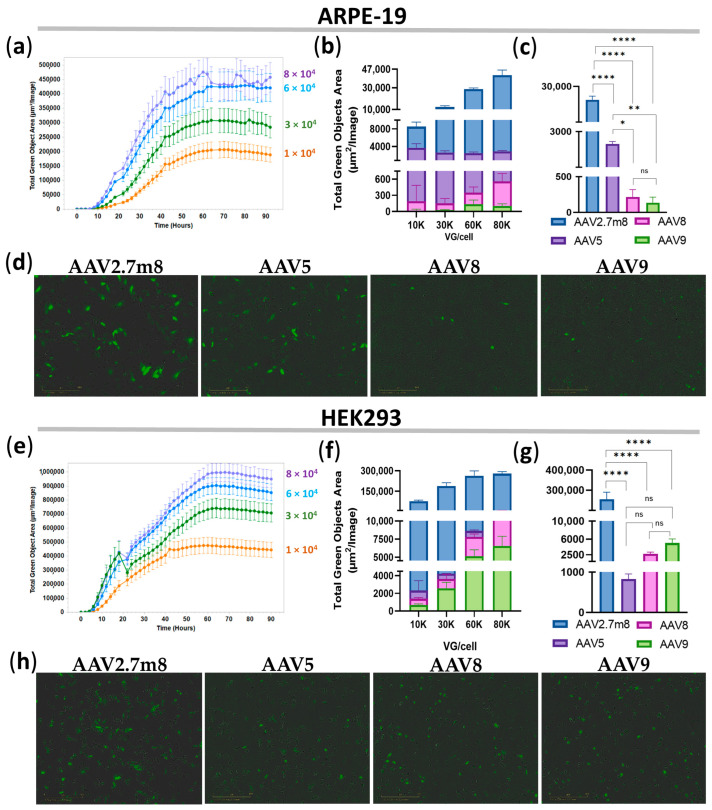
Superior transduction efficiency by AAV2.7m8 after 12 h. Transduction efficiency by the four AAV serotypes in ARPE-19 (**a**–**d**) and HEK293 (**e**–**h**) cells was assessed bi-hourly using an IncuCyte S3 live microscope with the intensity of GFP fluorescence presented by Total Green Objects Area (TGOA) values. (**a**,**e**) Dose-dependent GFP expression during the 4-day time course in ARPE-19 (**a**) and HEK293 (**e**) cells transduced by AAV2.7m8. Comparison of transduction efficiencies by the four serotypes (AAV2.7m8, AAV5, AAV8, and AAV9) at viral concentrations of 1-, 3-, 6-, and 8 × 10^4^ VG/cell (**b**,**f**) and statistical analysis between these serotypes at 6 × 10^4^ VG/cell in ARPE-19 (**c**) and HEK293 (**g**) cells. GFP fluorescence images in IncuCyte at 0.5 days post-viral transduction of ARPE-19 (**d**) and HEK293 (**h**) cells, respectively. (*) *p*-val < 0.05, (**) *p*-val < 0.01, (****) *p*-val < 0.0001, not significant (ns) *p* > 0.05.

Transduction of ARPE-19 and HEK293 cell lines with AAV2.7m8-, AAV5-, AAV8-, and AAV9-GFP at four concentrations: 1-, 3-, 6-, and 8 × 10^4^ VG/mL, demonstrated a dose-dependent increase in GFP fluorescence for all serotypes (Table S1), as exemplified by AAV2.7m8-transduced ARPE-19 (Figure 1a) and HEK293 (Figure 1e) cells. Notably, GFP expression as a result of AAV2.7m8 transduction of both cell lines was observed as early as 12 h (0.5 days) post-transduction at all viral concentrations and greatly exceeded that of serotypes AAV5, AAV8, and AAV9 in both ARPE-19 (Figure 1b,d) and HEK293 (Figure 1f,h) cells, for which the increase in fluorescence was observed more than 24 h post-transduction. Fluorescence intensity reached a plateau at 4.8 × 10^5^ TGOA in ARPE-19 cells (Figure 1a) and 1 × 10^6^ TGOA in HEK293 (Figure 1e) cells 48 h post-transduction, indicating that the transduction efficiency of HEK293 cells routinely used in in vitro AAV studies is on average twice as efficient as that of the human retinal pigment epithelial cells relevant to ophthalmogenetics studies. The average GFP fluorescence levels for all viral concentrations at 12 h post-transduction were 282, 81, and 281 times higher in ARPE-19 cells and 253, 92, and 52 times higher in HEK293 cells transduced by AAV2.7m8-GFP as compared to AAV5, AAV8, and AAV9, respectively (Table S1), and they were somewhat similar between the higher doses of 6 × 10^4^ and 8 × 10^4^ VG/mL. Specifically, the transduction efficiency of AAV2.7m8 at a viral dose of 6 × 10^4^ VG/cell was 21, 202, and 323 times higher in ARPE-19 cells and 324, 100, and 52 times higher in HEK293 cells than that of AAV5, AAV8, and AAV9, respectively. Statistical analysis of TGOA values obtained for the 6 × 10^4^ VG/mL viral dose at this time point showed that the level of fluorescence intensity in ARPE-19 (Figure 1c) and HEK293 (Figure 1g) cells transduced by AAV2.7m8 was significantly different [*p*-value (*p*) < 0.0001] compared to the other three serotypes, while AAV5 showed differences compared to AAV8 (*p* < 0.05) and AAV9 (*p* < 0.01). Meanwhile, no significant differences were observed between AAV5, AAV8, and AAV9 (*p* > 0.05) in HEK293 cells.

### 3.3. The Higher Fluorescence Intensity in AAV2.7m8-Transduced Cells Remains for 4 Days

**Figure 2:**
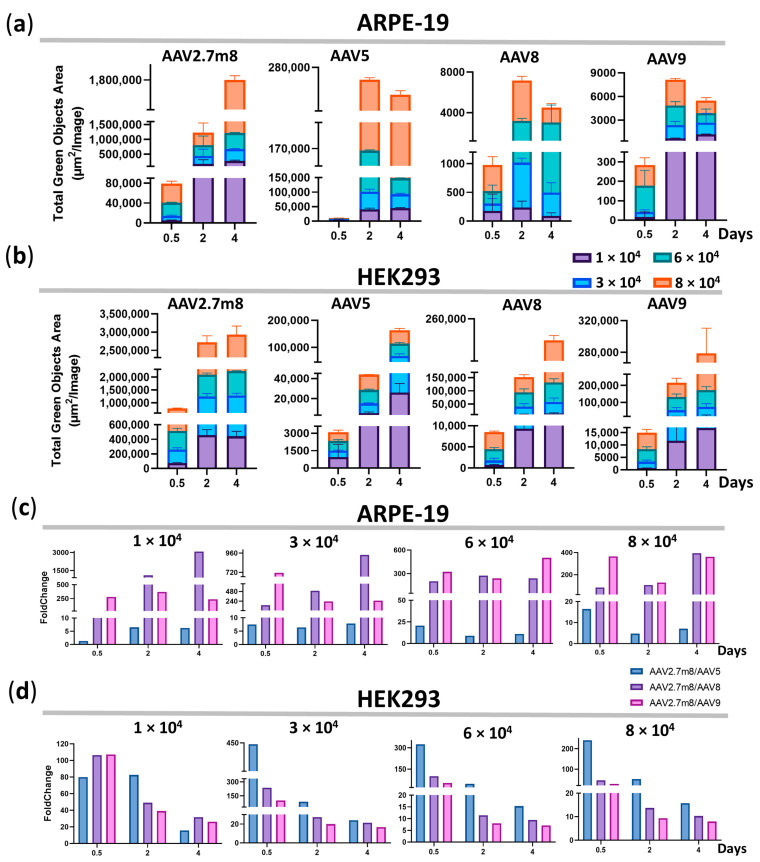
The higher fluorescence intensity in AAV2.7m8-transduced cells remains for 4 days. GFP fluorescence intensity [Total Green Objects Area (µm2/image) (TGOA)] values at 0.5, 2, and 4 days for the four serotypes and the fold change differences between AAV2.7m8 and AAV5, AAV8, or AAV9 in ARPE-19 (**a**,**c**) and HEK293 (**b**,**d**) cells.

The difference in GFP fluorescence levels in ARPE-19 and HEK293 cells transduced by AAV2.7m8 and other serotypes remained at all viral concentrations and time points. The maximum average fluorescence levels reached 579.1 × 10^3^, 3295, 3983, and 3294 TGOA in ARPE-19 cells, and 944.3 × 10^3^, 49.3 × 10^3^, 82.2 × 10^3^, and 106.6 × 10^3^ in HEK293 cells for AAV2.7m8, AAV5, AAV8, and AAV9, respectively (Figure 2a,b, Table S1). This is reflected in the fold change differences between AAV2.7m8 and other serotypes (Figure 2c,d) at all viral concentrations at the three time points. Overall, the level of GFP fluorescence as a result of AAV2.7m8 transduction of ARPE-19 cells throughout the course of analysis at four viral concentrations was 1.3–20.6, 25.5–3083, and 128.6–714.2 times higher than that of serotypes AAV5, AAV8, and AAV9 (Table S2).

Compared to the second-most efficient transducing serotype AAV5 in ARPE-19 cells, the GFP expression level post-AAV2.7m8 transduction was 21, nine, and 11 times higher at 0.5, 2, and 4 days, respectively, at 6 × 10^4^ VG/cell. Overall, the fold change difference between these serotypes averaged five at the minimum concentration of 1 × 10^4^ VG/cell and nine at the highest concentration of 8 × 10^4^ VG/cell throughout the course of analysis (Figure 2c and Table S2); however, the maximum difference was observed at 12 h post-transduction at 6 × 10^4^ VG/cell. Throughout the time course, AAV5 was five, seven, 13, and nine times less efficient as compared to AAV2.7m8, while the average fold change difference between AAV2.7m8 and AAV8 was 1403, 526, 239, and 195; AAV2.7m8 and AAV9 were 295, 401, 354, and 285 at 1-, 3-, 6-, and 8 × 10^4^ VG/cell, respectively.

Not entirely unexpectedly, the second-most effective HEK293-transducing serotype was AAV9, which is not used in clinical trials for gene delivery to retinal cells. It outperformed AAV5 and AAV8, despite the fact that AAV8 is frequently used in clinical trials. Throughout the course of analysis, AAV9 was 57, 45, 22, and 15 times less efficient as compared to AAV2.7m8 (Figure 2d and Table S2), while the average fold change difference between AAV2.7m8 and AAV5 was 59, 185, 128, and 103, and between AAV2.7m8 and AAV8–62, 95, 40, and 23 at 1-, 3-, 6-, and 8 × 10^4^ VG/cell, respectively. The lowest fold change difference of seven was observed between AAV2.7m8 and AAV9 at 6–8 × 10^4^ VG/cell on day 4.

### 3.4. The Trend Remains until the End, as Demonstrated by Flow Cytometry

**Figure 3:**
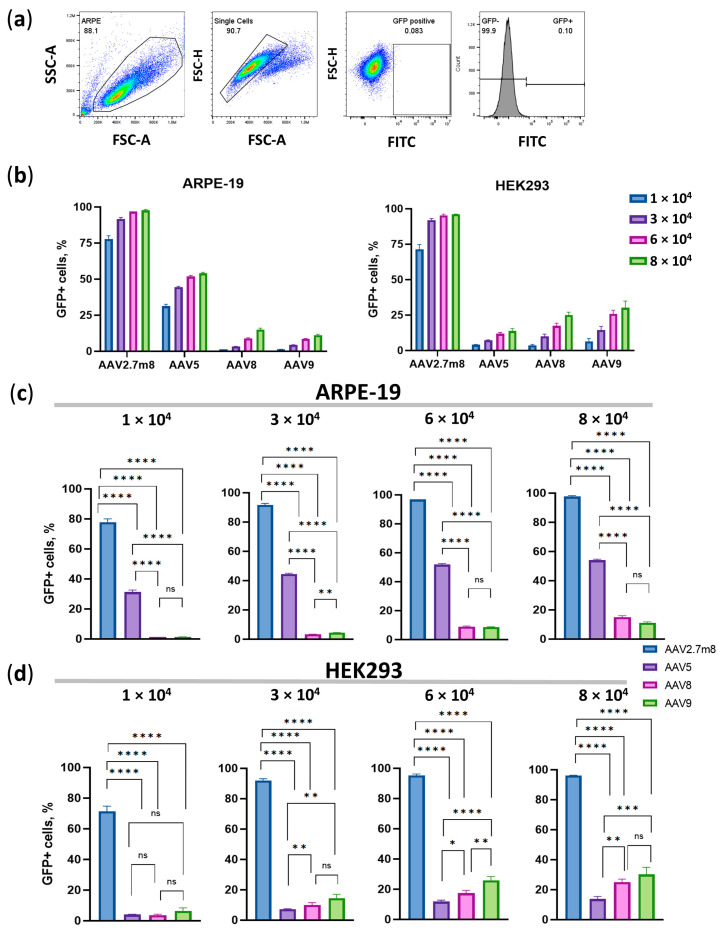
Flow cytometry analysis on day 4 post-transduction with the four AAV serotypes. (**a**) Gating strategy. Dose-dependent percentage of GFP-positive (GFP +) ARPE-19 and HEK293 cells (**b**) and statistical analysis of GFP+ ARPE-19 (**c**) and HEK293 (**d**) cell populations transduced with the four serotypes at four viral concentrations. Statistically significant differences (*p* < 0.0001) were observed between all compared serotypes at all concentrations, with the exception of AAV8 and AAV9 (*p* > 0.05) in ARPE-19 cells. A high level of statistical significance (*p* < 0.0001) at all viral concentrations was demonstrated in HEK293 cells transduced by the three serotypes as compared to AAV2.7m8, and by AAV5 as compared to AAV9 (*p* < 0.001) and AAV8 (*p* < 0.01) at high viral concentrations (6- and 8 × 10^4^ VG/cell). (*) *p*-val < 0.05, (**)–*p*-val < 0.01, (***) *p*-val < 0.001, (****) *p*-val < 0.0001, not significant (ns)–*p* > 0.05.

Ninety-six hours post-transduction, the number of GFP-positive cells and their median fluorescence intensity (MFI) values were assessed by flow cytometry. Untransduced live single HEK293 cells (the gating strategy is shown in Figure 3a) were used as a negative fluorescence control to set the gate for the GFP-positive population.

The overall trend, demonstrated earlier by means of IncuCyte data, remained as the number of GFP+ AAV2.7m8-transduced ARPE-19 cells was about 95% at both 6- and 8 × 10^4^ VG/cell (Figure 3b,c and Table S3) while only 50% of AAV5- and 10% of AAV8- and AAV9-transduced cells were GFP+ at these viral concentrations. In HEK293 cells, 28%, 21%, and 13% of AAV9, AAV8, and AAV5-transduced cells were GFP+ as compared to 96% of AAV2.7m8-transduced cells. Statistically significant differences (*p* < 0.0001) were observed between all compared serotypes at all concentrations, with the exception of AAV8 and AAV9 (*p* > 0.05) in ARPE-19 cells (Figure 3c). A high level of statistical significance (*p* < 0.0001) at all viral concentrations was demonstrated in HEK293 cells transduced by the three serotypes compared to AAV2.7m8, and by AAV5 compared to AAV9 (*p* < 0.001) and AAV8 (*p* < 0.01) at high viral concentrations (6- and 8 × 10^4^ VG/cell) (Figure 3d). It was evident that the concentration of 6 × 10^4^ VG/cell is the lowest viral dose that allows for significant statistical differences when AAV5 is compared to AAV8 and AAV9, while even the highest dose is insufficient to observe significant differences between AAV8 and AAV9. At a dose of 6 × 10^4^ VG/cell, AAV2.7m8 (97% GFP+ cells) was nearly twice and 10 times as efficient as AAV5 (52% GFP+ cells) and AAV9 or AAV8 (both 9%), respectively, in ARPE-19 cells. 95% of HEK293 cells transduced by AAV2.7m8 were GFP+, followed by AAV9- (26%), AAV8- (17%), and AAV5- (12%)-transduced cells.

**Figure 4:**
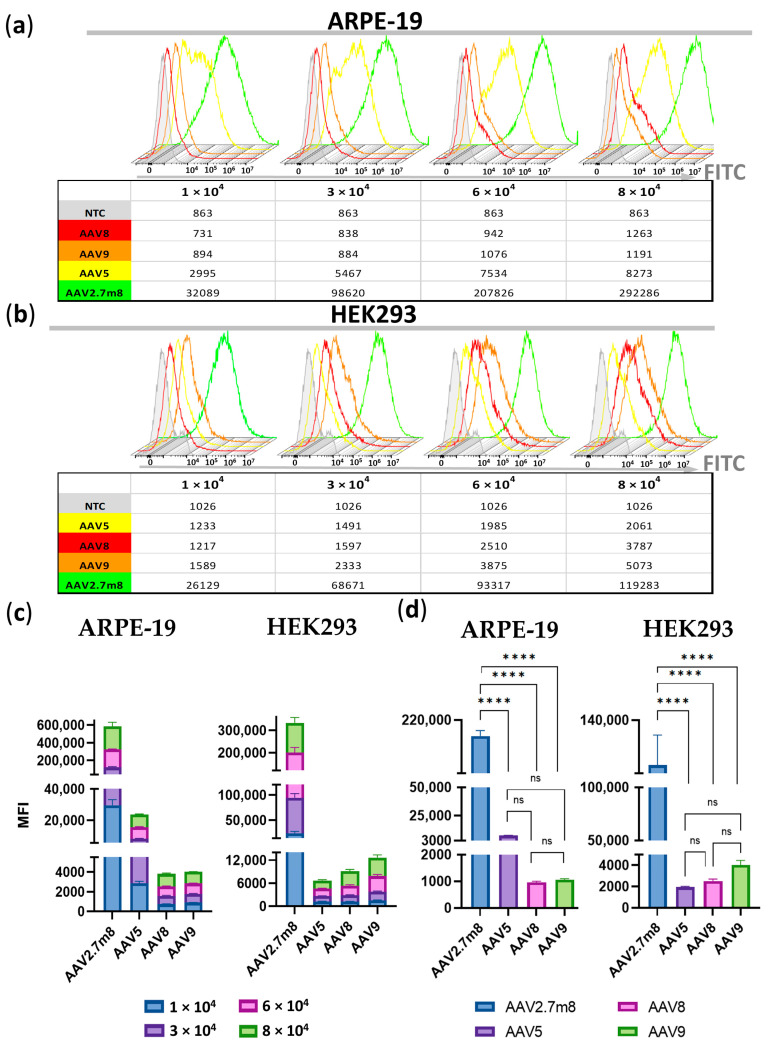
Significantly higher MFI (median fluorescence intensity) values in ARPE-19 (**a**) and HEK293 (**b**) cells transduced by AAV2.7m8 compared to AAV5, AAV8, and AAV9 at the four viral concentrations demonstrated by the histogram overlays. (**c**) Fold change differences between MFIs obtained for the four viral concentrations of each of the compared serotypes in ARPE-19 and HEK293 cells. (**d**) Statistical analysis of MFI values between AAV serotypes at 6 × 10^4^ VG/cell: the differences between AAV2.7m8 and the three serotypes were statistically significant (*p* < 0.0001) while no significant differences were found between AAV5-, AAV8-, and AAV9-transduced ARPE-19 and HEK293 GFP-positive populations (*p* > 0.05). (****) *p*-val < 0.0001, not significant (ns)–*p* > 0.05.

A dose-dependent increase in MFI values was observed for all serotypes in ARPE-19 and HEK293 cells (Figure 4a,b, respectively, and Table S4). The average MFI values for all viral concentrations in AAV2.7m8-transduced ARPE cells (146410) were 25 (5936), 154 (952), and 151 (972) times higher, and in HEK293 cells 50 (1677), 37 (2280), and 26 (3165) times higher, than those of AAV5, AAV8, and AAV9, respectively (Figure 4c).

While no significant differences were found between AAV5-, AAV8-, and AAV9-transduced ARPE-19 and HEK293 GFP+ populations (*p* > 0.05), the differences between AAV2.7m8 and the three serotypes were statistically significant (*p* < 0.0001) at 6 × 10^4^ VG/cell (Figure 4d).

At a dose of 6 × 10^4^ VG/cell, the average MFI of AAV2.7m8-transduced ARPE-19 GFP-positive cells (204 × 10^3^) was 28 and 193 times higher than that of AAV5 (7375) and AAV9 or AAV8 (1055 and 962), respectively, while in AAV2.7m8-transduced HEK293 cells (106 × 10^3^), these values were 26, 43, and 54 times higher than those of AAV9 (4016), AAV8 (2485) and AAV5 (1950 MFI), respectively (Table S4).

## 4. Discussion

AAV-based gene replacement therapy is a promising approach for the therapy of monogenic diseases, especially those caused by loss-of-function mutations. The selection of optimal serotypes based on their tropism permits targeted gene delivery to specific tissues and individual cells of the host. The ability of specific AAV serotypes to transduce the target cells, such as human retinal cells, depends on their tropism and transduction efficiency, which directly affects the success of IRD gene replacement therapy, for example. For this purpose, natural serotypes such as AAV2 are used as the basis for the production of new, optimized synthetic serotypes that demonstrate high, improved tropism towards specific cells. The AAV2 serotype was successfully used for the treatment of LCA2 (Luxturna), and the AAV5 and AAV8 serotypes have previously demonstrated high tropism to retinal cells. Two new synthetic serotypes, AAV7m8 and AAV8BP2, have been tested for their ability to transduce mouse and primate retinas. AAV2.7m8 efficiently transduces a variety of cell types when injected subretinally, whereas transduction after intravitreal delivery is limited [15]. The self-complementary viral vectors scAAV1, scAAV2, scAAV5, and scAAV8 were also tested on ARPE-19 and 661W cell lines; scAAV1 and scAAV2 were the most efficient in both cell lines, with Y-F capsid mutations increasing transduction efficiency [17]. An efficient transgene delivery to photoreceptors in mice and non-human primates leading to retinal restoration was achieved with the help of AAV2.7m8, a modified synthetic variant developed by in vivo-directed evolution [14]. This serotype includes 10 amino acids in loop 4 of the capsid protein structure, which forms a binding domain interacting with the heparin receptor. The LALGETTRPA peptide was inserted at position R588, located in the variable region of the AAV2 serotype VIII (VR-VIII) capsid [19]. This modification has been shown to disrupt the binding of AAV2 to its major receptor, heparan sulfate proteoglycan (HSPG) [14]. The reduced binding of rAAV2.7m8 to HSPG is hypothesized to promote vector distribution in the retina, in contrast to natural AAV serotypes such as AAV2 [14,19,20]. This serotype has shown efficient retinal transduction and strong transgene expression in small and large animal models after intravitreal delivery, which is less invasive than the traditional subretinal administration that can be harmful to the structure and function of the retina, especially if the patient’s retina is in the process of degeneration [21].

This study focused on exploring the tropism and transduction efficiency of the natural serotypes AAV5, AAV8, and AAV9, and the synthetic AAV2.7m8, delivering the GFP reporter gene into the retinopathy-relevant ARPE-19 and the popular conventional HEK293 cell models. The additional aim of the study was to determine and compare the dose-dependent transduction by these serotypes; therefore, the research was focused on the four viral concentrations: 1-, 3-, 6-, and 8 × 10^4^ VG/cell. The intensity of GFP expression was measured every 2 h using IncuCyte S3 live imaging microscopy. As demonstrated in Figure 1, AAV2.7m8 exhibited the highest transduction efficiency as compared to other serotypes at 12 h post-transduction. At 6 × 10^4^ VG/cell, AAV2.7m8 was 20, 201, and 323 times more efficient in ARPE-19 cells and 324, 99, and 52 times more efficient in HEK293 cells as compared to AAV5, AAV8, and AAV9, respectively. This trend remained in both ARPE-19 and HEK293 cell lines for 96 h (Figure 2). The superiority of AAV2.7m8 was further confirmed by flow cytometry analysis carried out at 96 h post-transduction (Figure 3 and Figure 4).

In 2018, Duong et al. compared eleven AAV serotypes: the naturally occurring serotypes 1–9 and the modified serotypes AAV2.7m8 and 8b, in human iPSC, iPSC-derived RPE, iPSC-derived cortical, and dissociated embryonic day 18 rat cortical neurons [16]. All recombinant AAVs delivered the eGFP transgene. Transduction was performed at three viral concentrations: 1 × 10^4^, 1 × 10^5^, and 1 × 10^6^ VG/cell, and GFP expression was measured 24, 48, 72, and 96 h post-transduction. While all serotypes showed measurable levels of AAV-eGFP expression, AAV7m8 and AAV6 demonstrated the highest transduction efficiency [16], which is in agreement with our data in ARPE-19 and HEK293 cell models. Considering that the high viral doses may induce a strong immune response, our study concentrated on the more fine-tuned dose-dependent viral transduction at concentrations of 1-, 3-, 6-, and 8 × 10^4^ VG/cell and demonstrated the high efficiency of AAV2.7m8-induced transduction at 6 × 10^4^ VG/cell. The data obtained by the live imaging microscopy, performed bi-hourly for 4 days, identified the rate of transduction by different serotypes. 

In conclusion, our study compared the transduction efficiency of the four AAV serotypes: AAV2.7m8, AAV5, AAV8, and AAV9, expressing GFP, and demonstrated that AAV2.7m8 displays superior qualities in two models: HEK293 and ARPE-19, the more relevant retinal pigment epithelium cell line, in terms of the rate of transduction of both HEK293 and ARPE-19 cells.

## Data Availability

The data can be shared upon request.

## Supplementary Materials

The following supporting information can be downloaded at: https://www.mdpi.com/article/10.3390/pharmaceutics16010138/s1, Figure S1: Schematic presentation of the virus production, purification, transduction and analysis; Figure S2: Graphical presentation of the affinity chromatography process; Figure S3: Sample analysis by means of the dynamic light scattering (DLS) method; Figure S4: Sample analysis by means of size-exclusion chromatography (SEC) method; Figure S5: Transmission electron microscopy (TEM) of the virus preparations. Table S1: Intensity of GFP fluorescence presented as Total Green Objects Area (µm^2^/image); Table S2: Fold change differences between AAV2.7m8 and other serotypes at different viral concentrations on days 0.5, 2 and 4; Table S3: The number of GFP+ cells at 96 h post transduction as demonstrated by flow cytometry analysis; Table S4: Median fluorescence intensity (MFI) of GFP+ populations.
