# Supplementary material for: Engineered AAV2.7m8 Serotype Shows Significantly Higher Transduction Efficiency of ARPE-19 and HEK293 Cell Lines Compared to AAV5, AAV8 and AAV9 Serotypes
Source: Pharmaceutics. 2024 Jan 19;16(1):138. doi: 10.3390/pharmaceutics16010138 (PMC10818700; doi:10.3390/pharmaceutics16010138)
Supplement: Supplementary file 1 [file pharmaceutics-16-00138-s001.zip › Figure S1.pdf]

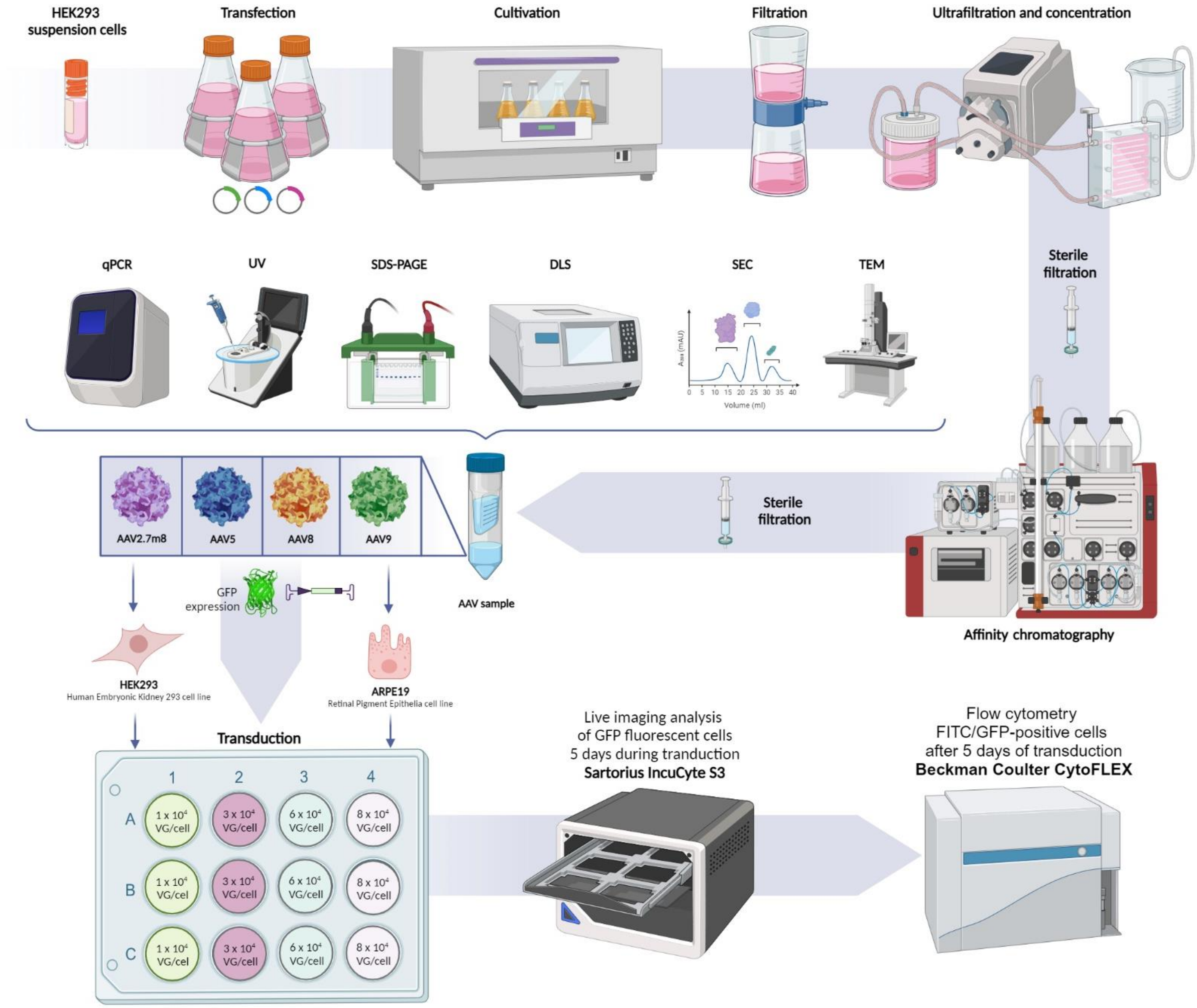

Figure S1. Schematic presentation of the virus production, purification, transduction and analysis.
