# Supplementary material for: Engineered AAV2.7m8 Serotype Shows Significantly Higher Transduction Efficiency of ARPE-19 and HEK293 Cell Lines Compared to AAV5, AAV8 and AAV9 Serotypes
Source: Pharmaceutics. 2024 Jan 19;16(1):138. doi: 10.3390/pharmaceutics16010138 (PMC10818700; doi:10.3390/pharmaceutics16010138)
Supplement: Supplementary file 1 [file pharmaceutics-16-00138-s001.zip › Figure S2.pdf]

AAV8-GFP

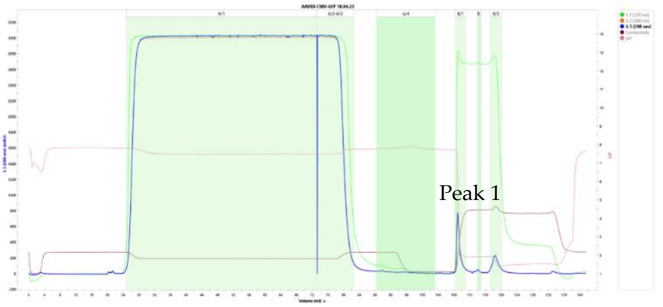

AAV5-GFP

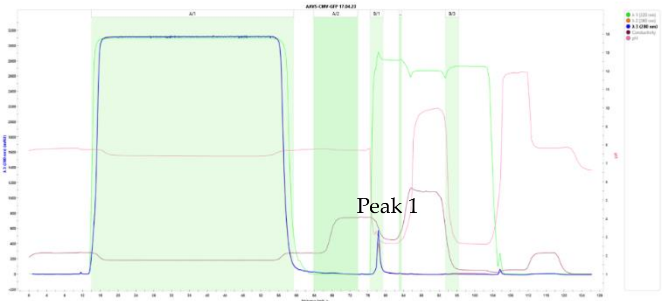

AAV9-GFP

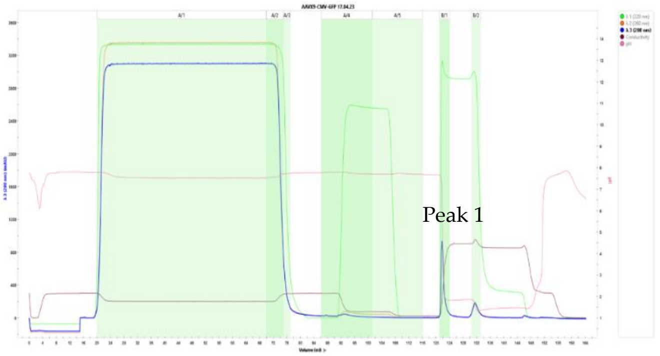

AAV2.7m8-GFP

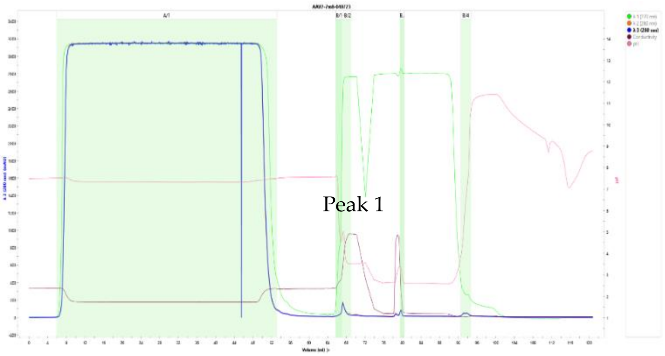

Figure S2. Graphical presentation of the affinity chromatography process. Peak 1 – elution of the virus. The fractions were obtained after elution with the respective buffers and the pH of the eluate was adjusted to 7.0.
