# Supplementary material for: Engineered AAV2.7m8 Serotype Shows Significantly Higher Transduction Efficiency of ARPE-19 and HEK293 Cell Lines Compared to AAV5, AAV8 and AAV9 Serotypes
Source: Pharmaceutics. 2024 Jan 19;16(1):138. doi: 10.3390/pharmaceutics16010138 (PMC10818700; doi:10.3390/pharmaceutics16010138)
Supplement: Supplementary file 1 [file pharmaceutics-16-00138-s001.zip › Figure S3.pdf]

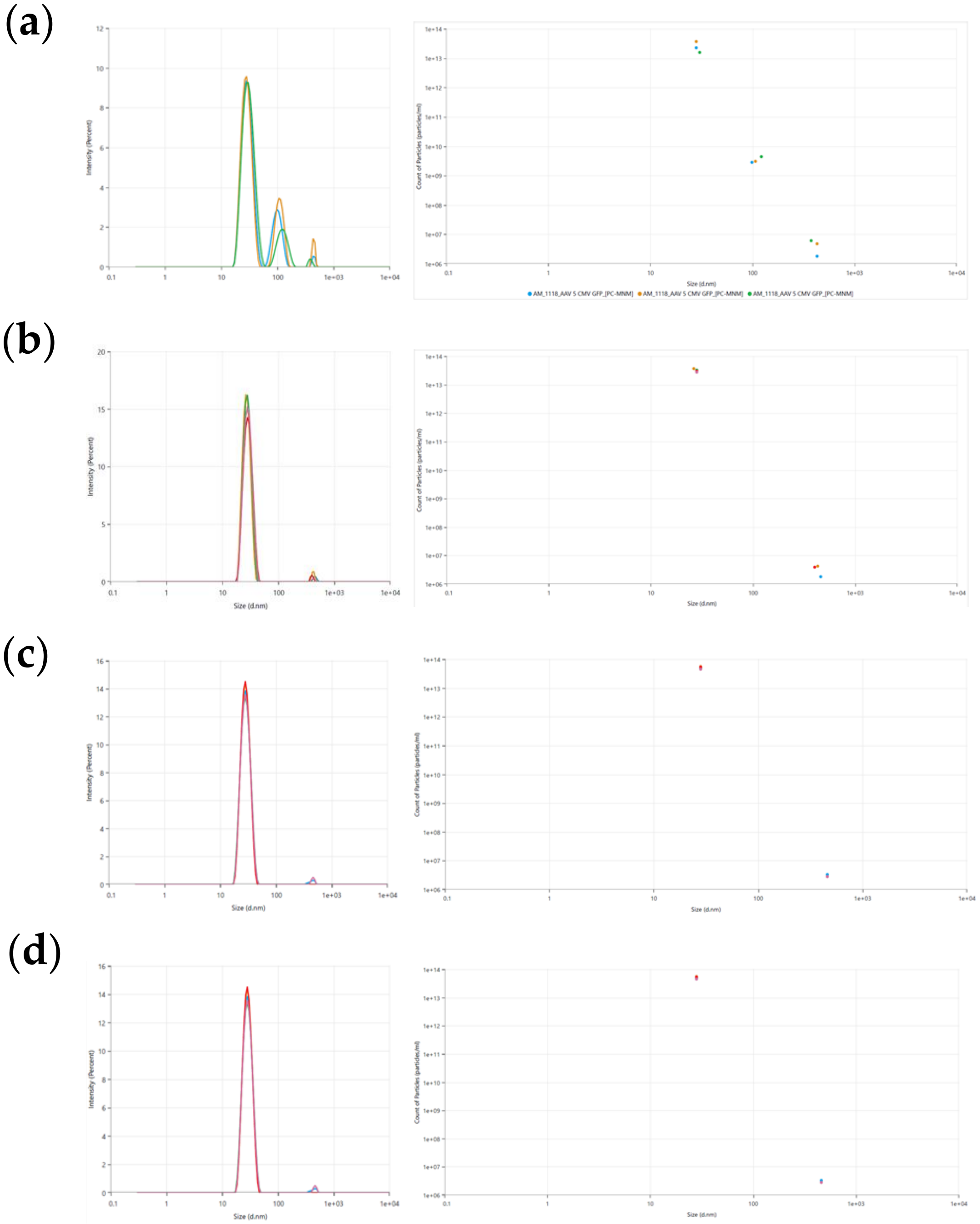

Figure S3. Sample analysis by dynamic light scattering (DLS) method. Particle size distribution plots of the AAV samples in relation to particle intensity and concentration. **(a)** – AAV5-GFP, **(b)** – AAV8-GFP, **(c)** – AAV9-GFP, **(d)** – AAV2.7m8-GFP. The obtained results indicate the presence of AAV particles with a hydrodynamic diameter of 29,6 nm, 28.3 nm, 28.8 nm and 26.6 nm in the samples at a concentration of 2,5x10^{13}, 3.21x10^{13}, 5.01x10^{13} and 8.4x10^{11} for AAV5-GFP, AAV8-GFP, AAV9-GFP and AAV2.7m8-GFP, respectively. The average volume fraction of particles is 99.99%.
