# Supplementary material for: Engineered AAV2.7m8 Serotype Shows Significantly Higher Transduction Efficiency of ARPE-19 and HEK293 Cell Lines Compared to AAV5, AAV8 and AAV9 Serotypes
Source: Pharmaceutics. 2024 Jan 19;16(1):138. doi: 10.3390/pharmaceutics16010138 (PMC10818700; doi:10.3390/pharmaceutics16010138)
Supplement: Supplementary file 1 [file pharmaceutics-16-00138-s001.zip › Figure S4.pdf]

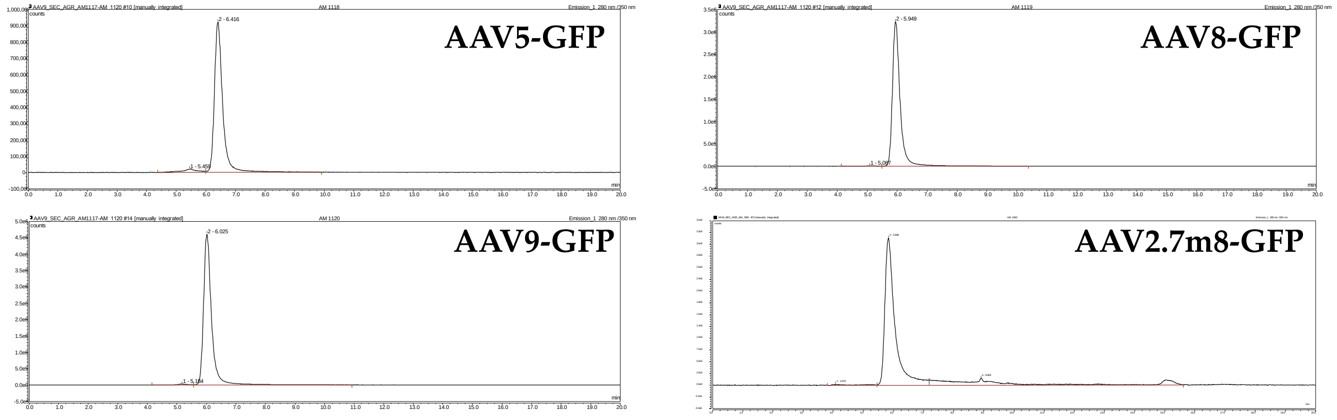

Figure S4. Sample analysis by size-exclusion chromatography (SEC) method. The relative monomer percentage for AAV5-GFP, AAV8-GFP, AAV9-GFP and AAV2.7m8-GFP samples was 95,9%, 99,4%, 99,2% and 79,8%; the aggregate fraction was 4,1%, 0.6%, 0,8% and 0,9%, respectively.
