# Supplementary figures and images for: Engineered AAV2.7m8 Serotype Shows Significantly Higher Transduction Efficiency of ARPE-19 and HEK293 Cell Lines Compared to AAV5, AAV8 and AAV9 Serotypes
Source: Pharmaceutics. 2024 Jan 19;16(1):138. doi: 10.3390/pharmaceutics16010138 (PMC10818700; doi:10.3390/pharmaceutics16010138)

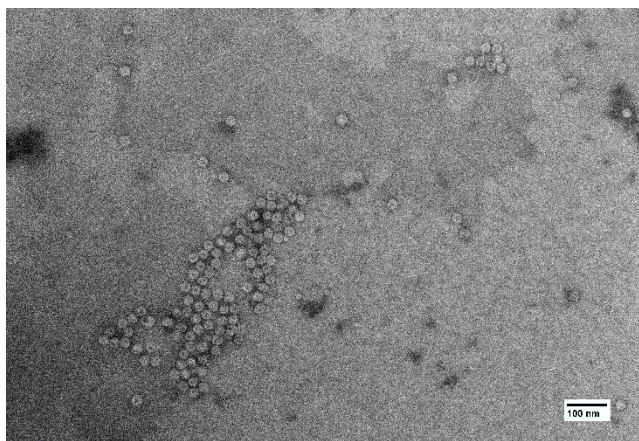

**AAV2.7m8**

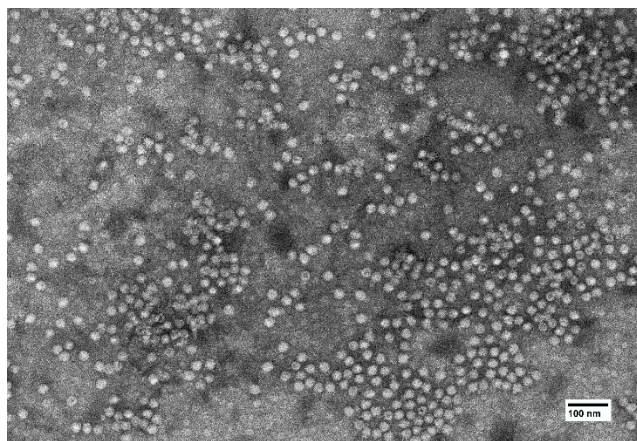

**AAV5**

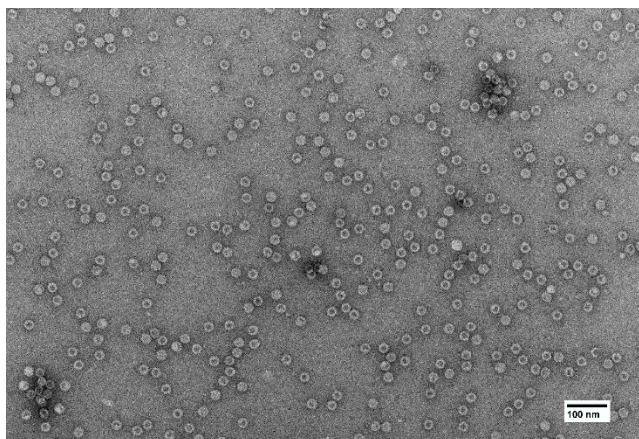

**AAV8**

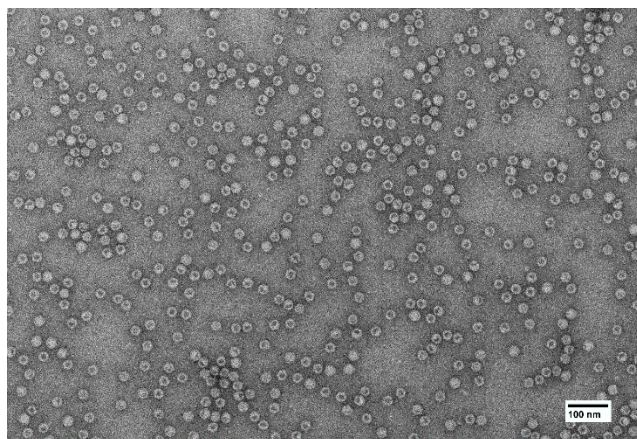

**AAV9**

**Figure S5.** Transmission electron microscopy (TEM) of the virus preparations.

Supplement: Supplementary file 1 [file pharmaceutics-16-00138-s001.zip › Figure S5.pdf]
